# 445. COVID-19 Pharmacotherapy Was Not Associated with Mortality in a Community Teaching System

**DOI:** 10.1093/ofid/ofab466.644

**Published:** 2021-12-04

**Authors:** Eric Urnoski, Thomas Butler

**Affiliations:** Crozer-Chester Medical Center, Havertown, Pennsylvania

## Abstract

**Background:**

During the COVID-19 pandemic, a task force was assembled to collect data on patient characteristics and treatment exposures to assess what factors may contribute to patient outcomes, and to help develop institutional treatment guidelines.

**Methods:**

A retrospective study was performed on COVID-19 inpatient admissions within a four-hospital community health system over a six-month period from April-October 2020. Positive COVID-19 immunology results and/in conjunction with an inpatient admission was criteria for inclusion. Covariates for age, gender, race were added *apriori.* Covariates of interest included baseline comorbidities, admission level-of-care, vital signs, mortality outcomes, need for intubation, and specific pharmacological treatment exposures. Logistic regression was performed on our final model and reported as OR +/- 95% CI.

**Results:**

A total of 349 patients met inclusion criteria. Pharmacotherapies were not associated with a difference in mortality in a four-hospital system. Corticosteroids (p = 0.99); Remdesivir (p = 0.79); hyrdroxychloroquine (p = 0.32); tocilizumab (p = 0.91); were not associated with mortality. ACE-inhibitor or angiotensin II receptor blockers OR 0.29 (0.09-0.93) (p = 0.03); convalescent plasma OR 7.85 (1.47-42.1) (p = 0.02); neuromuscular blocking agents (NMBA) OR 5.51 (1.28-23.8) (p = 0.02); vasopressors OR 17.6 (5.62-54.9) (p = 0.00) were associated with in-hospital mortality. Covariates that were associated with a difference in mortality were: age > 60 years OR 2.73 (1.04-7.14) (p = 0.04); structural lung disease OR 3.02 (1.28-7.10) (p = 0.01). Covariates not associated with mortality included African American race (p = 0.30); critical care admission (p = 0.19); obesity (p = 0.06); cardiovascular disease (p = 0.89); diabetes (p = 0.28).

**Conclusion:**

The use of corticosteroids, remdesivir, tocilizumab, and hydroxychloroquine, and admission to a critical care bed was not associated with a difference of in-hospital mortality. Patients who required vasopressors or NMBA were associated with in-hospital mortality. Despite national trends reporting increased mortality in patients with obesity, diabetes, cardiovascular disease, and of African American race, this was not observed in our health system safety net hospitals.

**Disclosures:**

**All Authors**: No reported disclosures

